# The Association Between Heat Stress and Child Stunting, Wasting, and Underweight Under Varying Vegetation Covers in Ethiopia

**DOI:** 10.1111/mcn.70227

**Published:** 2026-07-28

**Authors:** Daniel Abera Denssa, Desalew Meseret Moges, Bedasa Tessema, Feyissa Challa, Meseret Woldeyohannes, Alemnesh Petros, Meron Girma, Alemayehu Hussen, Abel Woldetinsae, Yetayesh Maru, Stanley Chitekwe, Ramadhani Noor, Mesay Hailu, Getachew Tollera, Mandana Arabi, Shilpa Rao, Carl Lachat, Eleni Papadopoulou, Masresha Tessema

**Affiliations:** ^1^ Ethiopian Public Health Institute Addis Ababa Ethiopia; ^2^ Department of Green Chemistry and Technology Ghent University Gent Belgium; ^3^ United Nations Children's Fund (UNICEF) Addis Ababa Ethiopia; ^4^ Research and Development, Nutrition International Ottawa Ontario Canada; ^5^ Global Health Cluster, Norwegian Institute of Public Health Oslo Norway; ^6^ Department of Food Technology, Safety and Health Ghent University Gent Belgium

**Keywords:** children under five, heat stress, nutritional status, Universal Thermal Climate Index, vegetation index

## Abstract

Climate change has significantly contributed to rising malnutrition rates, particularly in low‐ and middle‐income countries. However, evidence linking heat stress to child nutritional status remains limited. This study estimated the association between heat stress exposure and severe stunting, wasting and underweight among rural children across areas with varying vegetation density. We used data from Ethiopia's Food and Nutrition Strategy Baseline Survey, a population‐based cross‐sectional study conducted between 2021 and 2023. Our study population included a total of 7166 children aged 0–59 months. We estimated environmental exposures to heat stress and green areas using satellite‐based data on Universal Thermal Climate Index (UTCI) and the Normalized Difference Vegetation Index (NDVI). We used multilevel logistic regression models to assess associations between heat stress and severe stunting, wasting and underweight, adjusting for child age, maternal education, family size, and household wealth. About 23% of children (*n* = 1541) were severely stunted, 5.4% (*n* = 443) were identified with severe wasting and 8.4% (*n* = 761) with severe underweight. Among children living in low‐vegetation areas (NDVI < 0.44), exposure to heat stress was associated with higher odds of severe wasting (AOR = 1.63, 95% CI = 1.06–2.51) and severe underweight (AOR = 1.63, 95% CI = 1.20–2.22) compared with children not exposed to thermal heat stress conditions. Conversely, in areas of moderate‐to‐high vegetation covers (NDVI ≥ 0.44), heat stress was associated with a 28% decrease in odds of severe stunting (AOR = 0.72, 95% CI = 0.52–0.99). Our findings call on policymakers to implement climate‐resilient agriculture, and enhance social protection to safeguard livelihoods and food systems in vulnerable, low‐resource settings.

## Summary

- Peak heat stress intensity and potential maximum exposure duration (in hours) vary across the different administrative regions of Ethiopia.
- In Ethiopia vegetation might modify the impact of heat stress on child nutritional outcomes.
- Children who lived in low‐vegetation cover areas had a higher likelihood of experiencing severe wasting and severe underweight when exposed to heat stress.
- Satellite‐based UTCI and NDVI are informative tools for identifying climate‐related nutrition risks.

## 1 Introduction

The threat of climate change to food security involves the breakdown of food systems, including crops and livestock, as well as interruptions in food distribution (Godde et al. 2021; Mirzabaev et al. 2023). Additionally, climate change can reduce the diversity of available foods, leading in inadequate dietary diversity, which is crucial for children's growth and development (Mannar et al. 2020; Owino et al. 2022). This food shortage, particularly during the crucial early years of life, contributes to child undernutrition (Belesova et al. 2019).

Children are more vulnerable than adults to climate‐related health threats, such as diarrheal diseases (Weeda et al. 2024), mainly because of their rapid metabolism and developing systems (Anderko and Pennea 2022; Randell et al. 2020; Van der Merwe et al. 2022). More than 1 billion children or nearly half the world's children, are at extremely high risk of ongoing and worsening exposure to climate and environmental shocks (UNICEF 2018). Climate change and extreme weather events have largely driven a marked increase in child undernourishment since 2015 (Niles et al. 2021; Onyango et al. 2019; Van der Merwe et al. 2022).

Indicators of malnutrition in children such as stunting and wasting, are significant risk factors for childhood mortality, with malnutrition accounting for approximately 45% of deaths among children under five (Black et al. 2013; Olofin et al. 2013). Severe wasting is a deadly type of undernutrition. It accounts for nearly 20% of fatalities among children under 5 years old, and impacts 13.7 million children globally each year (Locks et al. 2025). Survivors of childhood undernutrition often suffer lasting consequences, including impaired health, reduced cognitive development and productivity, and increased vulnerability to non‐communicable diseases in adulthood (Global Nutrition Report 2021; Victora et al. 2021).

The risks associated with climate‐related heat impacts are anticipated to be more pronounced in already hot environments that lack sufficient resources for adaptation (Godde et al. 2021). Low‐ and middle‐income countries (LMICs) face the greatest risks from climate change, despite contributing relatively low levels of greenhouse gas emissions (Naser et al. 2024) because of limited resources, inadequate planning, and insufficient investment in healthcare, environmental protection, and related sectors (Mazumder and Hossain 2024). In sub‐Saharan Africa, the situation is particularly severe, as the region is home to one‐third of all malnourished children globally (Baker and Anttila‐Hughes 2020; Njatang et al. 2023; Onyango et al. 2019). A comprehensive study across 18 countries in sub‐Saharan Africa, revealed that high temperatures and low rainfall negatively affect the nutritional status of children aged 0–59 months, reducing weight and increasing the risk of wasting, underscoring their vulnerability to climate variability (Thiede and Strube 2020). Higher temperatures observed during the survey month, the year leading up to the survey, and throughout the child's lifetime contributed to significant declines in measures of child nutritional status (Baker and Anttila‐Hughes 2020). Nevertheless, the combined effects of temperature, humidity, and wind speed on child nutrition remain largely unexplored and poorly understood.

This study aims to fill critical gaps in understanding how heat stress affects child nutritional outcomes in rural Ethiopia. We employ the Universal Thermal Climate Index (UTCI), a comprehensive measure of heat stress that integrates multiple environmental factors, to assess its association with severe wasting, stunting and underweight at the individual household level, which has not previously been conducted in this context. Furthermore, we examine whether these associations vary across vegetation density levels, as measured by the Normalized Difference Vegetation Index (NDVI). By integrating high‐resolution environmental data with individual‐level child nutritional status, this study advances current knowledge on the pathways linking climate‐related heat exposure and child undernutrition in low‐resource settings.

## 2 Methods

### 2.1 Study Setting

**Figure 1:**
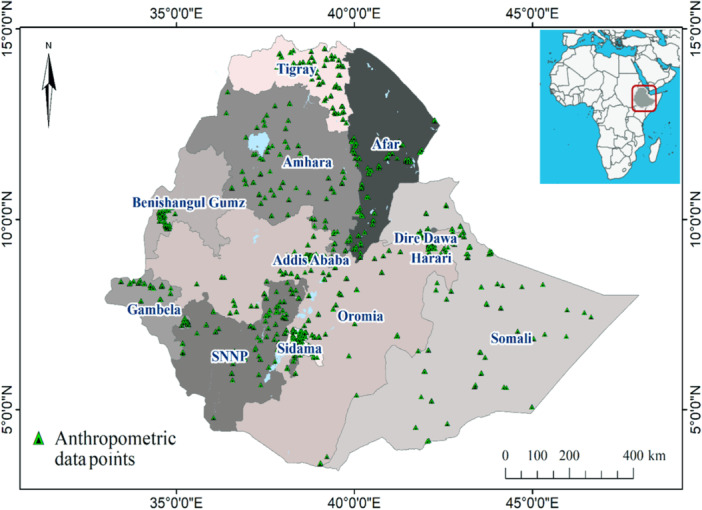
Map of Ethiopia showing administrative regions and locations of households where child anthropometric measurements were collected.

The study was conducted in Ethiopia, focusing on the rural areas of Oromia, Amhara, Tigray, Sidama, Gambela, Afar, Somali, Benishanguel Gumz, Hareri region, Dire Dawa City administration, and SNNPR (i.e., SNNPR has been further divided into three newly established regions, namely, Southwest Ethiopia Peoples' Region, Central Ethiopia Regional State, and South Ethiopia Regional State). Ethiopia's climate is shaped by altitudinal differences and seasonal rainfall, with distinct wet and dry periods influenced by the Intertropical Convergence Zone. Temperatures vary from about 10°C in the highlands to over 47°C in the arid lowlands. It also has diverse vegetation types, including moist and dry evergreen Afromontane forests in the highlands, Combretum‐Terminalia and Acacia‐Commiphora woodlands in the lowlands, savanna grasslands, and desert scrublands (Asefa et al. 2020). The nation's diverse ecosystems support a variety of livelihoods, primarily agriculture and livestock rearing. Agriculture contributes 40% to the country's GDP (World Bank 2021). Ethiopia has a population of around 126.5 million, 70% of whom live in rural areas (WorldBank 2023). Furthermore, based on UN‐derived age distribution data, children aged 0–59 months constitute about 13.5% of the total population, corresponding to roughly 17 million (United Nations 2024), reflecting an upward trend from the roughly 13 million children reported by the CSA in 2007 (CSA 2007). Figure 1 offers a geographical representation of the study areas and study households.

### 2.2 Study Design and Population

Data were sourced from the nationally and sub‐nationally representative Ethiopia's Food and Nutrition Strategy Baseline Survey (FNS) conducted between 2021 and 2023. FNS's assessed the anthropometric status, dietary intake, and micronutrient status of Ethiopian children, women of reproductive age, and adolescent girls. It used a stratified two‐stage sampling design. Additional details on the FNS study, its design and sampling procedures were presented elsewhere (Woldeyohannes et al. 2023).

Our study population comprised children aged 0–59 months. Among the 10, 991 children participating in the FNS, we included only those from rural areas (*n* = 7166, 65%). In this study, rural areas follow the classification of the Central Statistical Agency (CSA) of Ethiopia, which defines them as localities without municipal administration or administrative functions, typically comprising fewer than 2000 inhabitants, with most residents engaged in agricultural activities (CSA 2007). In addition, participants with missing or implausible information on child age, height, or weight were excluded from the respective analyses. After applying these exclusion criteria, the final analytic samples included 6644 children for stunting, 6610 for wasting, and 6902 for underweight. We focus on rural areas because, in urban settings, heat exposure is additionally influenced by the urban heat island effect, surface characteristics, and anthropogenic heat emissions, which interact with vegetation in complex ways and can differ substantially from rural conditions (Huang et al. 2016).

### 2.3 Study Variables

The outcome variables were severe stunting, wasting and underweight. The anthropometric measurements were taken following the standard procedures outlined by the World Health Organization (WHO 2019). Namely, children with severe stunting are those with a height‐for‐age z‐score more than three standard deviations below the WHO Child Growth reference median, children with severe wasting are those with a weight‐for‐height z‐score more than three standard deviations below the WHO median, and children with severe underweight are those with a weight‐for‐age z‐score more than three standard deviations below the WHO median. All anthropometric indices were calculated using the WHO Anthro software to generate standardized growth measures (WHO 2009). These Z scores were calculated using the Child Growth Standards (De Onis et al. 2012). Anthropometric data were collected by a trained data collector who also attended the measurement standardization exercise. All clusters and individual data were geo‐referenced.

Our independent variables were a climate variable, UTCI and a climate‐related environmental variable, NDVI. UTCI is a comprehensive measure of thermal comfort that incorporates multiple meteorological factors, including temperature, humidity, wind speed, and solar radiation, to provide an in‐depth assessment of outdoor thermal conditions. Hourly UTCI data from ERA5‐HEAT (spatial resolution: 0.25° × 0.25°) were extracted for 10,991 survey locations from 2015 to 2024, covering the gestational period corresponding to children who were aged 0–59 months at the start of data collection in 2021. It was computed using NetCDF, terra, and sf packages in R. From these values, we derived two key thermal exposure indicators: Mean Annual Lifetime UTCI (Malt‐UTCI), calculated as the average UTCI from 1 year before birth to the year of interview, representing a child's cumulative exposure to heat stress; and a 90‐day average UTCI (UTCI‐90) preceding the interview date, capturing short‐term exposure to heat stress conditions. We then categorized the Malt‐UTCI and UTCI‐90 values according to the standard UTCI thermal stress classification (Błażejczyk et al. 2013), including the categories of extreme cold stress (below −40°C), Very strong cold stress (−27°C to −40°C), Strong cold stress (−13°C to −27°C), (0°C to −13°C) Slight cold stress (9°C to 0°C), no thermal heat stress (26°C to 9°C), moderate heat stress (32°C to 26°C), strong heat stress (38°C to 32°C), very strong heat stress (46°C to 38°C), and extreme heat stress (above 46°C). Furthermore, the total hours of exposure to various heat stress levels (UTCI‐90) were computed and presented as percentages, using the estimated maximum exposure duration of 2160 h (90 days × 24 h).

NDVI is a widely used remote sensing index that measures vegetation cover intensity by comparing near‐infrared (strongly reflected by vegetation) and red light (absorbed by vegetation). It serves as a proxy for ecosystem productivity, vegetation health, and agricultural conditions (Shabanov et al. 2002). NDVI data were obtained from the MODIS MOD13Q1 product, accessed via the Land Processes Distributed Active Archive Center (LP DAAC) website (https://lpdaac.usgs.gov/). This dataset consists of composite images with a spatial resolution of 250 meters. NDVI values for the main growing seasons in Ethiopia, June to September, were obtained for survey locations. For each child, NDVI was computed for the year of birth (n), gestational year (n‐1), and 2 years before birth (n‐2), following the same approach used in a previous work conducted in West Africa (Johnson and Brown 2014), resulting in a set of mean annual values which represented the vegetation density for the child's residence.

### 2.4 Statistical Analyses

We reported continuous variables as mean ± SD when normally distributed and as median and interquartile range (IQR) when non‐normally distributed, while frequencies and percentages were used for categorical variables. We conducted multilevel logistic regression analyses to quantify the association between heat exposure and the three child nutrition outcomes. Heat stress was measured using Malt‐UTCI for severe stunting and underweight, and UTCI‐90 for severe wasting. Multilevel logistic regression models accounted for both covariate effects and random effects of the enumeration area to capture geographical clustering of the data. Both unadjusted and adjusted models were fitted and presented. The results from unadjusted analyses are presented to show the crude associations, while adjusted models provide effect estimates accounting for confounding. Potential confounders were selected based on prior knowledge and available information, guided by a directed acyclic graph (DAG) developed for this study (Supporting Information S1: Figure S1). The adjustment set included child age categorized in six groups (0–5 months, 6–11 months, 12–23 months, 24–35 months, 36–47 months and 48– 59 months), maternal education level categorized as: no formal education, primary (1–8 years of education), secondary (9 to 12 years of education), and higher than secondary including college or university graduates, household wealth index categorized in three categories: poor (quantile 1 and quantile 2), middle (quantile 3), and rich (quantile 4 and quantile 5), household family size: five or less, and more than five, and enumeration area. We treated diarrhea and household food insecurity status as mediators based on the DAG. Their relationship with heat stress is presented separately in Supporting Information S1: Tables S4 and S5, respectively.

Households were assigned to pre‐established wealth classifications based on data from the Demographic and Health Surveys Wealth Index (Rutstein 2008). However, our three categories were defined to fit the distribution of our study population, since very small numbers belonged in the two extreme quintiles (1 and 5). Additionally, household food insecurity was estimated using the FAO Food Insecurity Experience Scale (FIES), which includes eight questions on self‐reported food‐related behaviors and experiences linked to growing challenges in accessing food due to resource constraints (Wambogo et al. 2018). We analyzed data on diarrheal episodes reported within the 2 weeks preceding the survey. Heat stress was modeled as a categorical variable in the multilevel regression analyses, based on the observed non‐linear relationship between heat stress and child nutritional outcomes (Supporting Information S1: Figure S5, panels A–C). In addition, the monthly distribution and variability of heat stress, and its relationship with the vegetation index, were illustrated using time series plots (Supporting Information S1: Figure S4A,B), including those for two heat stress hotspots in Ethiopia (Supporting Information S1: Figure S4C,D). Furthermore, a stratified analyses were conducted to assess potential heterogeneity in effect by vegetation cover, estimating associations between heat stress and child undernutrition separately for low‐ and moderate‐to‐high vegetation areas. The national mean NDVI value of 0.44 was used to define the strata, with low‐vegetation cover defined as NDVI < 0.44 and moderate‐to‐high vegetation cover defined as NDVI ≥ 0.44.

The results from multilevel logistic models were finally presented as adjusted odds ratios (AORs) with 95% confidence intervals (CIs). Statistical significance was set at a two‐tailed *p*‐value < 0.05. All analyses were conducted using STATA software, version 16.

### 2.5 Ethics Statement

This study was conducted in accordance with the guidelines laid down in the Declaration of Helsinki, and all procedures involving research participants were approved by the Institutional Review Board of the Ethiopian Public Health Institute (protocol no: EPHI‐IRB‐317–2020). Written informed consent was obtained from all subjects.

## 3 Results

### 3.1 Characteristics of the Study Population

The prevalence of severe stunting, wasting and underweight among pre‐school age children in rural residence of Ethiopia is presented in Table 1. Most of the children included in this study 62% (*n* = 4114) were 24–59 months old. More than half or 68% of their mothers (*n* = 3832) had no formal education, and approximately two‐thirds (70%) of the children reside in households within the poorest wealth quantiles. In addition, one‐third of them live in food‐insecure households. Within our study population in rural Ethiopia, 23% (*n* = 1541) of children aged 0–59 months were severely stunted, 5.4% (*n* = 443) were identified with severe wasting and 8.4% (*n* = 761) with severe underweight. The prevalence of severe stunting, wasting and underweight differs across child age, administrative regions and maternal education categories (*χ* ^2^ test, *p* < 0.001). Yet, there is a statistically significant relationship between household food security status and severe stunting or underweight, and between wealth status and severe wasting among children (*χ* ^2^ test, *p* < 0.05). A regional comparison showed that the Afar region in the northeast exhibited the highest prevalence of severe stunting (28.3%), severe wasting (12.2%), and severe underweight (7.7%). On the contrary, the prevalence of severe stunting is the lowest in the Gambela region situated in the western part of the country (Table 1). Additional details on the prevalence of three child nutrition outcomes, regardless of residence type, are presented separately (Supporting Information S1: Table S5).

**Table 1:** Weighted percentages of severe stunting, wasting and underweight among rural children aged 0–59 months.

		Children with severe stunting (HAZ < −3)				Children with severe wasting (WHZ < −3)				Children with severe underweight (WAZ < −3)		
	*N*	%	*n*	*p*‐value^a^	*N*	%	*N*	*p*‐value^a^	*N*	%	*N*	*p*‐value^a^
Child age in months				*p* < 0.001				*p* < 0.001				*p* < 0.001
< 6	809	9.7	76		788	13.7	96		898	4.9	54	
6–11	509	13.4	82		513	3.9	37		538	4.6	37	
12–23	1,212	24.2	300		1207	7.2	90		1247	7.2	130	
24–35	1,327	29.4	402		1330	4.4	81		1370	11.5	211	
36–47	1,466	26.6	383		1462	4.4	87		1499	10.7	187	
48–59	1,321	23.7	298		1310	2.1	52		1350	7.9	142	
Region				*p* < 0.001				*p* < 0.001				*p* < 0.001
Afar	773	28.3	231		763	14	112		799	23.9	201	
Amhara	608	22.0	131		594	8.8	55		618	7.7	49	
Benishangul‐Gumz	588	27.2	157		583	5.2	32		624	12.3	74	
Dire Dawa	276	27.5	79		275	6	13		292	10.7	34	
Gambela	423	12.6	50		423	7.3	31		429	7.3	29	
Harari	415	25.0	104		416	3.9	17		422	7.1	32	
Oromia	923	23.7	212		922	4.1	41		958	7.9	76	
Sidama	516	21.9	111		504	7.9	39		550	11.7	65	
SNNP	735	24.4	176		735	3.9	29		752	7.6	54	
Somali	884	23.0	200		894	5.0	43		955	11.4	103	
Tigray	503	18.2	90		501	5.9	31		503	8.4	44	
Maternal' education status				*p* < 0.001				*p* < 0.001				*p* < 0.001
No education	3832	25.9	973		3815	6.1	298		4005	10.8	541	
Primary	1922	20.4	398		1907	4.7	94		1984	5.4	142	
Secondary	531	18.0	93		525	4.2	21		542	4.0	39	
Higher than secondary	124	7.3	13		126	5.8	13		128	4.4	12	
Wealth Quantile				*p* < 0.001				*p* < 0.05				*p* < 0.001
Poor	4625	25.0	1121		4617	6.0	339		4812	10	595	
Middle	1475	22.7	333		1461	4.4	73		1531	7.0	132	
Rich	544	14.2	87		532	5.2	31		559	3.4	34	
Food insecurity (FI), household				*p* < 0.05				*p* < 0.001				*p* < 0.05
Severe FI	2367	26.2	587		2373	4.4	126		2445	9.5	277	
Moderate FI	1337	24.0	305		1327	4.8	116		1413	9.8	189	
Food secure	2810	20.1	610		2784	6.7	195		2907	6.8	279	
Total	6644	23.0	1541		6610	5.4	443		6902	8.4	761	

^a^ Chi‐square test (X^2^); *N* stands for number of children (unweighted), *n* represents weighted number of children × 1000 included for stunting, wasting or underweight within each subcategory of all variables.

### 3.2 Distribution of UTCI and NDVI

The highest Malt‐UTCI was observed in Gambela (28.7°C) followed by Afar (27.7°C) and the Somali region (24.31°C). Similarly, the highest UTCI‐90 was observed in Afar (32°C) followed by Gambela (31°C) and Somali region (28.4°C). On the contrary, the least Malt‐UTCI value of 17.2°C was observed in Sidama region in the southern part of Ethiopia which only increased to a UTCI‐90 of 19.2°C closer to the interview date (Table 2). Table 2 presented descriptive statistics for the independent variables, namely: Malt‐UTCI, UTCI‐90, and NDVI across all administrative regions of Ethiopia.

**Table 2:** Summary statistics of Malt‐UTCI, UTCI‐90 and NDVI across all regions of Ethiopia.

Region	Malt‐UTCI (°C)		UTCI‐90 (°C)				NDVI
	Mean	SD	Mean	SD	Heat stress hours (%)^a^	Mean	SD
Afar	27.7	1.78	32.5	7.89	1319 (61)	0.29	0.14
Amhara	18.7	3.66	21.6	10.4	597 (28)	0.47	0.09
Benishangul Gumz	24.2	1.82	29.2	6.64	683 (32)	0.62	0.07
Dire Dawa	17.2	0.98	21.2	9.91	515 (24)	0.39	0.09
Gambela	28.7	2.32	31.2	6.7	1129 (52)	0.66	0.07
Harari	19.4	0.43	26.4	7.32	577 (27)	0.45	0.06
Oromia	17.5	2.75	20.5	9.85	500 (23)	0.49	0.1
Sidama	17.2	1.05	19.2	8.5	369 (17)	0.43	0.1
SNNPR	20.1	3.46	24.5	8.43	662 (31)	0.46	0.1
Somali	24.3	3.65	28.4	8.87	899 (42)	0.26	0.08
Tigray	18.8	2.61	22.2	10.8	690 (32)	0.39	0.06
National	20.7	2.09	24.5	8.65	722 (33)	0.44	0.09

^a^ Maximum potential exposure duration to heat stress conditions (> 26°C) within a 90‐day period prior survey, measured in hours and additionally expressed as a percentage of total hours within 90 days.

The distribution of average exposure duration to various levels of heat stress conditions (UTCI‐90) varies across the different regions in Ethiopia. Notably, children in the Afar region had the highest exposure duration to heat stress conditions (UTCI‐90 > 26°C) that persist for 1319 h within 90‐day period (61%), followed by Gambela and Somali regions at 1129 h (52%) and 899 h (42%), respectively. Also, these regions exhibited very strong heat stress levels (38°C to 46°C), which persisted for 508, 166, and 79 h, respectively (Supporting Information S1: Table S2). Additionally, the Malt‐UTCI and exposure durations (in hours) to the various heat stress categories (UTCI‐90) across Ethiopia's administrative regions were presented using a box‐whisker plot (Supporting Information S1: Figure S2A,B), respectively.

The distribution of mean annual NDVI for the survey locations showed that regions in the western part of Ethiopia (Gambela and Benishangul) exhibit greater vegetation cover, with mean NDVI values exceeding 0.6. Conversely, the Afar and Somali regions in the east display lower vegetation cover, with mean NDVI values falling below 0.3 (Table 2). The other regions show moderate vegetation coverage, with NDVI values ranging between 0.4 and 0.6 (Supporting Information S1: Figure S3A).

Overall, only a slight decrease in mean NDVI value was observed as the year of birth (YB) approached, dropping from 0.45 in the two preceding years (gestation period (YB‐1) and YB‐2), to 0.44 for the YB (Supporting Information S1: Figure S3B). This trend was consistent across seven administrative regions, namely, Gambela, Benishanguel Gumuz, Oromia, Amhara, SNNP, Sidama, and Hareri. On the contrary, the mean NDVI on the remaining four regions slightly increased moving to the YB. Additional detail for NDVI across rural residences and within 3 years up to the year of birth is provided (Supporting Information S1: Table S3).

**Figure 2:**
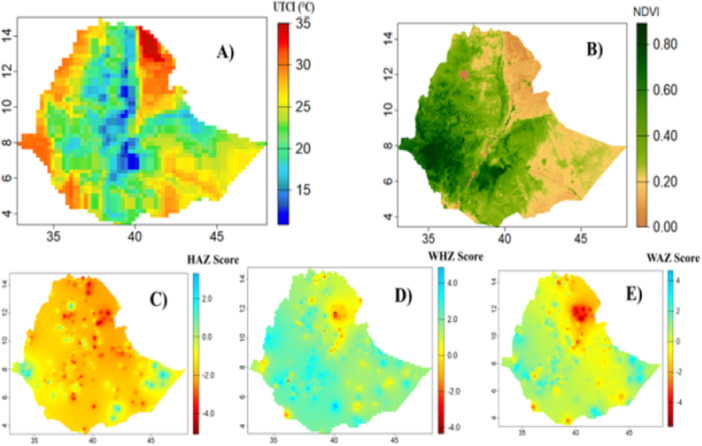
Spatial distribution of (A) Malt‐UTCI (2015–2024), (B) mean annual NDVI (2015–2024), (C) stunting, (D) wasting, and (E) underweight prevalence across the different regions of Ethiopia.

The spatial distribution of Malt‐UTCI and NDVI is presented along with the prevalence of stunting, wasting and underweight in Figure 2. The Malt‐UTCI map (Figure 2A) showed higher heat stress levels in the eastern part of Ethiopia, particularly in Afar region and the western part of the country: Gambela and Benishangul‐Gumuz regions while areas in the most central part of Ethiopia generally experiences no thermal heat stress. Areas that experience higher heat stress tend to have poorer vegetation cover in terms of NDVI, and areas with better vegetation tend to have lower heat stress levels (i.e., Malt‐UTCI). However, the Gambela region stands as one exception, where both high heat stress and high vegetation cover were observed (Figure 2A,B). Overall, it clearly illustrates that the WHZ score (Figure 2D) and WAZ score (Figure 2E) decreases in areas experiencing heat stress conditions combined with low‐vegetation cover.

### 3.3 Association Between Heat Stress and Severe Stunting, Wasting, and Underweight

Approximately, 19% (*n* = 1260) of children were potentially exposed to heat stress conditions throughout their lifetimes measured by Malt‐UTCI, regardless of their residence vegetation cover (Tables 3 and 5). Furthermore, during the 3‐month period prior to anthropometric data collection nearly, 24% (*n* = 1495) of children were likely exposed to heat stress conditions (Table 4). However, the duration of exposure in hours and peak heat stress level could vary across the different administrative regions of Ethiopia (Supporting Information S1: Table S2).

**Table 3:** Crude and adjusted associations between heat stress and severe stunting.

Crude model	*N*	%	OR (95% CI)	*p*‐value
No‐thermal stress (≤ 26°C) (reference)	5384	81.0		
Heat stress conditions (> 26°C)	1260	19.0	1.05 (0.86, 1.28)	0.61
Adjusted model^a^				
No‐thermal stress (≤ 26°C) (reference)	5384	81.0		
Heat stress conditions (> 26°C)	1260	19.0	0.99 (0.81, 1.23)	0.96
Stratified analyses				
Adjusted Model^a^ for low‐vegetation context (NDVI < 0.44)				
No‐thermal stress (≤ 26°C) (reference)	2816	80.1		
Heat stress conditions (> 26°C)	698	19.9	1.25 (0.95, 1.64)	0.11
Adjusted model^a^ for moderate‐to‐high vegetation context (NDVI ≥ 0.44)				
No‐thermal stress (≤ 26°C) (reference)	2568	82		0.04*
Heat stress conditions (> 26°C)	562	18	0.72 (0.52, 0.99)	

* Statistically significant (*p* < 0.05).

^a^ Adjusted for child age, maternal education, wealth status, and family size.

**Table 4:** Crude and adjusted associations between heat stress and severe wasting.

Crude model	*N*	%	OR (95% CI)	*p*‐value
No‐thermal stress (≤ 26°C) (reference)	4820	76.3		
Heat stress conditions (> 26°C)	1495	23.7	1.36 (0.97, 1.93)	0.07
Adjusted model^a^				
No‐thermal stress (≤ 26°C) (reference)	4820	76.3		
Heat stress conditions (> 26°C)	1495	23.7	1.32 (0.94, 1.87)	0.11
Stratified analyses				
Adjusted Model^a^ for low‐vegetation context (NDVI < 0.44)				
No‐thermal stress (≤ 26°C) (reference)	2683	76.8		
Heat stress conditions (> 26°C)	811	23.2	1.63 (1.06, 2.51)	0.03*
Adjusted model^a^ for moderate‐to‐high vegetation context (NDVI ≥ 0.44)				
No‐thermal stress (≤ 26°C) (reference)	2137	75.8		0.96
Heat stress conditions (> 26°C)	684	24.2	1.01 (0.58, 1.77)	

* Statistically significant (*p* < 0.05).

^a^ Adjusted for child age, maternal education, wealth status, and family size.

Multilevel logistic regression analyses indicated no clear association between heat stress and severe stunting in either the crude or adjusted models prior to stratification by NDVI (Table 3). In low‐vegetation areas (NDVI < 0.44), the adjusted odds ratio showed a slight upward trend, suggesting a possible increase in the likelihood of severe stunting with higher heat stress exposure, but the association was not clear. In contrast, among children residing in moderate‐to‐high vegetation areas (NDVI ≥ 0.44), the relationship appeared reversed, with higher heat stress associated with lower odds of severe stunting (AOR = 0.72; 95% CI: 0.52–0.99; *p* < 0.05). The crude and adjusted associations between heat stress and severe stunting, wasting and underweight are presented in Tables 3, 4, 5, respectively.

**Table 5:** Crude and adjusted associations between heat stress and severe underweight.

Crude model	*N*	%	OR (95% CI)	*p*‐value
No‐thermal stress (≤ 26°C) (reference)	5606	81.2		
Heat stress conditions (> 26°C)	1296	18.8	1.64 (1.27, 2.12)	0.000*
Adjusted model^a^				
No‐thermal stress (≤ 26°C) (reference)	5606	81.2		
Heat stress conditions (> 26°C)	1296	18.8	1.47 (1.14, 1.89)	0.003*
Stratified analyses				
Adjusted model^a^ for low‐vegetation context (NDVI < 0.44)				
No‐thermal stress (≤ 26°C) (reference)	2934	80.2		
Heat stress conditions (> 26°C)	723	19.8	1.63 (1.20, 2.22)	0.002*
Adjusted Model^a^ for moderate‐to‐high vegetation context (NDVI ≥ 0.44)				
No‐thermal stress (≤ 26°C) (reference)	2672	83.3		
Heat stress conditions (> 26°C)	573	17.7	1.19 (0.77, 1.84)	0.42

* Statistically significant (*p* < 0.05).

^a^ Adjusted for child age, maternal education, wealth status, and family size.

Consistent with the results for severe stunting, no clear association between heat stress and severe wasting was observed in either the crude or adjusted models prior to stratification by NDVI (Table 4). When adjusting for low‐vegetation, exposure to heat stress within 90 days prior to anthropometric measurement was associated with an increased odds of severe wasting (AOR = 1.63; 95% CI: 1.06–2.51; *p* < 0.05). In contrast, no association was observed between heat stress exposure and severe wasting among children residing in moderate‐to‐high vegetation areas (AOR = 1.01; 95% CI: 0.58– 1.77; *p* > 0.05).

Unlike the results of severe stunting and wasting, exposure to heat stress was associated with higher odds of severe underweight in all models, except for moderate‐to‐high vegetation setting. More specifically, in the crude model, exposure to heat stress conditions (Malt‐UTCI > 26°C) was associated with higher odds of severe underweight (AOR = 1.64; 95% CI: 1.27–2.12; *p* < 0.05). After adjustment for potential confounders the association was attenuated but remained statistically significant. Stratified analyses further revealed a stronger relationship in low‐vegetation areas (NDVI < 0.44) than in high‐vegetation areas, where exposure to heat stress was associated with 1.63‐fold higher odds of severe underweight (Table 5).

## 4 Discussion

This study examined the effect of heat stress experienced from in utero through early childhood on child nutritional outcomes in Ethiopia. We found a significant negative association between heat stress and severe stunting among children living in areas with moderate‐to‐high vegetation cover. For instance, although both Gambela in the west and Afar in the east experience high levels of heat stress, the higher NDVI in Gambela likely mitigates the heat stress impacts. Consequently, the Gambela region exhibited the lowest prevalence of severe stunting among rural residents in Ethiopia. Similar to our findings, a study in Burkina Faso and Mali reported strong responses to NDVI changes, where higher NDVI was associated with decreased odds of stunting in Mali (Johnson and Brown 2014). Also, both in Burkina Faso and Mali, higher NDVI was associated with a lower probability of mortality in children (Johnson and Brown 2014). Likewise, a recent study in the tropical highlands of Ethiopia, where nights are usually cold, found a decrease in stunting associated with exposures to higher temperatures during early life (Randell et al. 2020). In addition, a weak positive relationship between height‐for‐age and lifetime temperature exposure was reported in a study that combined anthropometric data from 38 sub‐Saharan African countries (Baker and Anttila‐Hughes 2020). Our study adds evidence for a plausible mechanism that explains the negative association between heat stress and severe stunting, described as the agricultural impact pathway. Under this mechanism, higher temperatures reduce the likelihood of frost and promote optimal vegetation growth, thereby contributing to improved food security (Holden et al. 2004).

On the contrary, we observed a weak, non‐significant positive trend between heat stress and severe stunting among children living in areas with low‐vegetation cover. Although the association did not reach statistical significance, the direction of the estimate suggests a potential relationship that may warrant further investigation. One possible explanation relates to the nonlinear decline in crop yields at higher temperatures, as highlighted in previous literature (Hatfield and Prueger 2015; Schlenker and Roberts 2009). In addition, children are particularly vulnerable to the direct effects of temperature shocks on health and become stunted, as they are unable to recognize and respond to heat stress independently (Zivin and Shrader 2016). Overall, our findings can offer an explanation for previously inconsistent results in the literature on the relationship between early‐life heat stress exposure and childhood stunting (Johnson and Brown 2014; Phalkey et al. 2015) by highlighting that the association between heat stress and stunting may be context‐dependent and potentially modified by vegetation conditions, as reflected by NDVI.

Regarding severe wasting, our findings suggest that in low‐vegetation areas, children exposed to heat stress are more likely to experience severe wasting, compared to those not exposed. However, no association was found among children who live in moderate‐to‐high vegetation areas. Similarly, low‐vegetation cover was associated with a higher prevalence of child wasting in Mali (Johnson and Brown 2014). In line with this, another study reported a 2.2 percentage‐point increase in the wasting rate due to 100 h of monthly exposure to temperatures between 30 and 35°C in the preceding 90 days (Blom et al. 2022). In our study, the highest prevalence of severely wasted children was reported in the Afar region (14%) where the average vegetation cover is very low, and it could be attributed to the prolonged exposure to high levels of heat stress (38°C to 46°C) over the preceding 90 days, totaling 508 h, or approximately 169 h per month. Such prolonged exposure to high heat stress levels may directly affect children's health by increasing their risk of dehydration, fever, and heat exhaustion, all of which contribute to child wasting (Zivin and Shrader 2016). Likewise, in a study by Bonell and her colleagues, increasing heat stress exposure was associated with reductions in weight‐for‐height and weight‐for‐age z‐scores among infants aged 12 months (Bonell et al. 2024). Furthermore, the link between heat stress and severe wasting may operate through health‐related pathways, as suggested by the observed relationship between heat stress and diarrheal illness, which increased under heat stress conditions, in line with the proposed causal framework.

In the present study, the prevalence of severe wasting in Gambela (7.3%) is lower than that of Afar, even though the exposure duration to heat stress is slightly lower, with children experiencing exposure to very strong heat stress in total for 166 h within 90 days or about 55 h per month. This lower prevalence of wasting may be partly explained by the region's relatively high vegetation cover, which could mitigate both the direct and indirect effects of heat stress on children's wasting. Supporting this is a study that reported that environmental greening reduced UTCI peaks by 6.9°C and shortened the duration of heat stress by 4.5 h (Huang et al. 2016), highlighting the protective role of vegetation in providing shade and reducing direct thermal exposure. Unlike previous studies that treated wasting in general, our analyses explicitly link severe wasting to extreme heat stress conditions in varying environmental settings measured by NDVI. Our findings demonstrate a distinct synergistic effect, providing evidence of how low‐vegetation cover, combined with peak heat stress, significantly increases the risk of severe wasting.

For underweight, we found that exposure to heat stress conditions is positively associated with severe underweight among children living in areas with low‐vegetation cover. This aligns with findings from previous studies conducted in Africa, in which low NDVI values were associated with higher rates of underweight (Johnson and Brown 2014; Njatang et al. 2023). In addition, higher vegetation cover has been associated with reduced malnutrition in other regions of Ethiopia (Njatang et al. 2023). However, cross‐country analyses revealed some inconsistencies in the observed relationship between vegetation density and child nutrition outcomes (Grace et al. 2022; Johnson and Brown 2014). Also, consistent with severe wasting, the link between heat stress and severe underweight may operate through health‐related pathways such as diarrheal illness. Our approach allowed us to clearly establish an association between heat stress and severe malnutrition indicators, an outcome that many similar studies have either failed to demonstrate or have reported inconsistently (Headey and Venkat 2024).

This study provides important evidence of the association among heat stress exposure, vegetation cover, and child malnutrition, also making our findings relevant for current policies. More specifically, recent reports indicate that over 85% of Ethiopia's land has experienced degradation to varying extents, with about 23% identified as degradation hotspots, areas that have been most severely affected over the past three decades (Gebreselassie et al. 2015). Such areas, characterized by soil degradation resulting from water erosion and the loss of vegetation cover, particularly in areas subjected to exploitative use (Nyssen et al. 2004), are largely a consequence of deforestation. This process leads to declining soil fertility, reduced moisture‐holding capacity, and, consequently, lower crop yields per hectare (Petr et al. 2010). Moreover, the absence of effective land‐use policies and corresponding legislation has further accelerated deforestation in Ethiopia, estimated at approximately 73,000 hectares per year, thereby exacerbating food insecurity in the country (FAO 2020). Consequently, strengthening coherence among forestry, agriculture, and nutrition policies remains essential to sustain the gains achieved in combating malnutrition while safeguarding ecosystem services. In this regard, the Ethiopian Green Legacy Initiative, introduced in 2019, is a groundbreaking reforestation project with the goals of preventing deforestation, repairing degraded landscapes, and boosting environmental resilience (Massrie 2024).

Our study methodology has several strengths, including the use of the Ethiopian Food and Nutrition Strategy (FNS) baseline survey, which provides nationally and sub‐nationally representative data. The study's broad geographic coverage across all regions of Ethiopia, characterized by diverse climatic zones, provides a unique opportunity to examine how heat exposure relates to child malnutrition across varied environmental contexts. Although extrapolation of the findings should be made with caution, the broad national scope enhances the relevance of the results for understanding climate‐nutrition relationships in heterogeneous settings. In addition, our study examined nutrition outcomes which are characterized by diverse and complex risk factor mechanisms, providing a comprehensive picture of child undernutrition in relation to climatic stressors. The anthropometric data collection was conducted according to international standards by trained data collectors, minimizing bias due to outcome misclassification. Finally, we linked individual‐level anthropometric data with high‐resolution environmental indicators that capture exposures during key developmental windows, using multilevel logistic regression models to account for data clustering across administrative levels, and we were able to provide detailed information on the important role of vegetation cover. This methodology provided high‐quality evidence to support elucidating the association between heat stress and child undernutrition, given the inconsistencies in the current literature in similar settings. However, even after adjusting for key confounders, unmeasured factors may still have influenced the observed associations. Additionally, the relatively coarse spatial resolution of the environmental data (~27 km) may have smoothed local heterogeneity, potentially introducing exposure misclassification, particularly in areas with diverse land cover or microclimates. Although restricting the analyses to rural areas and using average exposure values over relevant time periods likely reduced this bias, some degree of exposure misclassification may remain.

In conclusion, our study provides evidence of an association between early‐life heat stress exposure and severe malnutrition among rural Ethiopian children, contributing to a more coherent body of epidemiological findings within an otherwise inconsistent literature. Our findings indicate that children in rural areas, particularly those residing in regions where vegetation cover is below the national average, are more vulnerable to the effects of heat stress. This vulnerability appears to operate through multiple pathways, including agricultural, health‐related, and direct exposure mechanisms. These findings highlight the need for localized strategies that address both the immediate and long‐term consequences of climate change, and with a particular focus on heat stress. Priority actions include strengthening infrastructure and promoting regenerative, climate‐resilient agricultural practices such as agroforestry, drought‐resistant crop varieties, and soil and water conservation measures. Broader system‐level interventions within the food system are also essential. Furthermore, the government should prioritize strengthening social capital and building adaptive capacity by sustaining large‐scale tree‐planting initiatives, especially in degraded areas. Such efforts will support national goals related to climate change mitigation, sustainable agriculture, reduced undernutrition, and improved rural livelihoods.

## Author Contributions

Daniel Abera Denssa, Desalew Meseret Moges, Shilpa Rao, Carl Lachat, Eleni Papadopoulou, and Masresha Tessema conceptualized the study; Daniel Abera Denssa, Bedasa Tessema, Feyissa Challa, Meseret Woldeyohannes, Alemnesh Petros, Meron Girma, Alemayehu Hussen, Mesay Hailu, Getachew Tollera, and Masresha Tessema conducted research; Daniel Abera Denssa analyzed the data with help from Desalew Meseret Moges, Alemayehu Hussen, and Bedasa Tessema; Daniel Abera Denssa, Desalew Meseret Moges, Abel Woldetinsae, Carl Lachat, Eleni Papadopoulou, and Masresha Tessema wrote the paper. All authors took responsibility for reviewing, final editing, and approval of the manuscript.

## Conflicts of Interest

The authors declare no conflicts of interest. The opinions and statements in this article are those of the authors and may not reflect the official policies or opinions of the organizations they belong to.

## Supporting information


**Figure S1:** Directed acyclic graph (DAG) illustrating the potential confounding effects of child age, maternal education, family size, household wealth index; and mediators: food insecurity and diarrhea on the association between heat stress and child undernutrition under different vegetation cover (NDVI).
**Figure S2:** (A) Boxplots of mean annual UTCI (ºC) from 2015 to 2024 by region and (B) total hours of exposure to different heat stress levels over the 90‐day period preceding the data collection date, stratified by region.
**Figure S3:** A) Mean annual NDVI by region, and B) NDVI for the year of birth and the two preceding years by region.
**Figure S4‐A and Figure S4‐B:** Time series plots of mean Temperature (°C), UTCI (°C) and NDVI in Ethiopia (2015 to 2024).
**Figure S4‐C and Figure S4‐D:** Time series plots of mean Temperature (°C), UTCI (°C) and NDVI in heat stress hotspots (2015–2024).
**Figure S5:** A scatter plot with a local polynomial smoother showing the relationship between heat stress (x‐axis) and child nutrition status (y‐axis) Panels A–C, and between vegetation index (x‐axis) and child nutrition status (y‐axis) Panels D–F.

**Table S1:** Percent distribution of stunting, wasting and underweight among children aged 0–59 months in Ethiopia.
**Table S2:** A descriptive summary of exposure duration to different heat stress levels across all regions during the three months preceding anthropometric data collection.
**Table S3:** Descriptive summary of NDVI for all regions of rural residence within 3 years up to year of birth.
**Table S4:** Adjusted associations between heat stress and diarrheal cases.
**Table S5:** Adjusted associations between heat stress and food insecurity.

## Data Availability

The FNS datasets we used to generate anthropometric indices are available from the National Data Management Center (NDMC) of the Ethiopian Public Health Institute upon reasonable request. Access can be requested through the appropriate online procedures at the institutional website or by contacting the first author, subject to an embargo period to allow for the completion of ongoing analyses and publication of findings. Hourly Universal Thermal Climate Index (UTCI) data derived from the ERA5‐HEAT (Human thErmAL comforT) dataset were downloaded from the Copernicus Climate Data Store (https://cds.climate.copernicus.eu/). Daily temperature data from ERA5 (ECMWF/ERA5_LAND/DAILY_AGGR) and daily NDVI from the MODIS product (MODIS/MCD43A4_006_NDVI) were accessed through the Google Earth Engine (GEE) data catalog. The R scripts and GEE code used for climate data extraction, processing, and visualization are publicly available at: https://github.com/desalewm/climate_nutrition.
