# Supplementary material for: The Association Between Heat Stress and Child Stunting, Wasting, and Underweight Under Varying Vegetation Covers in Ethiopia
Source: Matern Child Nutr. 2026 Jul 28;22(3):e70227. doi: 10.1111/mcn.70227 (PMC13409331; doi:10.1111/mcn.70227)
Supplement: Supplementary file 1 — Figure S1: Directed acyclic graph (DAG) illustrating the potential confounding effects of child age, maternal education, family size, household wealth index; and mediators: food insecurity and diarrhea on the association between heat stress and child undernutrition under different vegetation cover (NDVI). Figure S2: (A) Boxplots of mean annual UTCI (ºC) from 2015 to 2024 by region and (B) total hours of exposure to different heat stress levels over the 90‐day period preceding the data collection date, stratified by region. Figure S3: A) Mean annual NDVI by region, and B) NDVI for the year of birth and the two preceding years by region. Figure S4‐A and Figure S4‐B: Time series plots of mean Temperature (°C), UTCI (°C) and NDVI in Ethiopia (2015 to 2024). Figure S4‐C and Figure S4‐D: Time series plots of mean Temperature (°C), UTCI (°C) and NDVI in heat stress hotspots (2015–2024). Figure S5: A scatter plot with a local polynomial smoother showing the relationship between heat stress (x‐axis) and child nutrition status (y‐axis) Panels A–C, and between vegetation index (x‐axis) and child nutrition status (y‐axis) Panels D–F. [file MCN-22-e70227-s002.docx]

**Supplementary Material 2:**


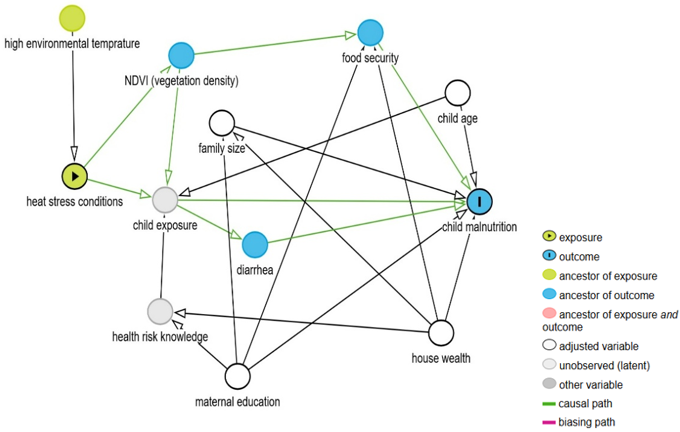


**Figure S1:** Directed acyclic graph (DAG) illustrating the potential confounding effects of child age, maternal education, family size, household wealth index, and the potential mediating effect of diarrhea and food insecurity on the association between heat stress and child undernutrition in varying environmental conditions expressed using Normalized Difference Vegetation Index (NDVI).


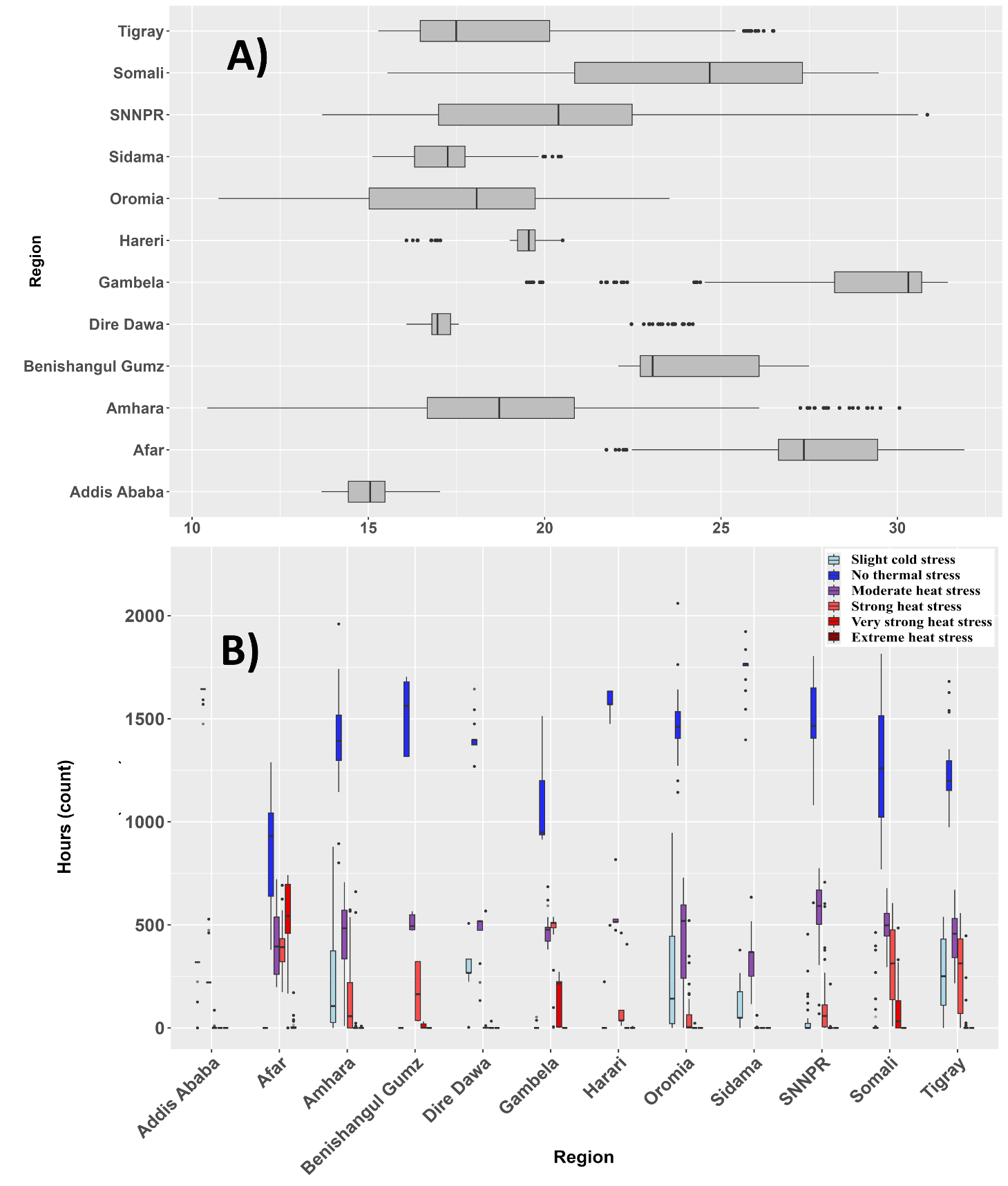


**Figure S2:** (A) Boxplots of Mean Annual Lifetime-Universal Thermal Climate Index (Malt-UTCI, ºC) from 2015 to 2024 by region and (B) duration of exposure (in hours) to different heat stress levels over the 90-day period preceding the data collection date, stratified by region.


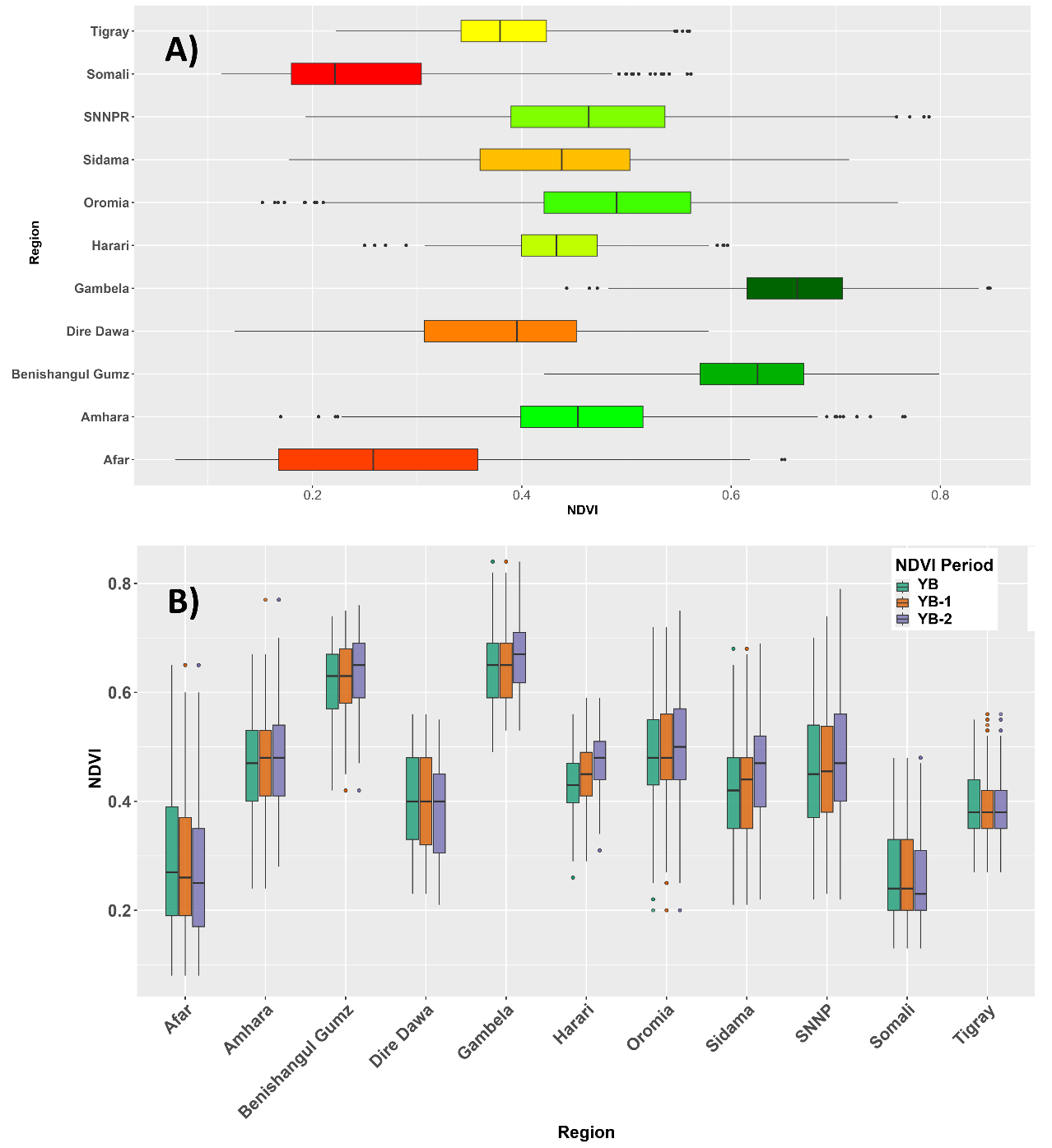


**Figure S3:** (A) Mean annual Normalized Difference Vegetation Index (NDVI) across regions, and (B) NDVI values during the year of birth (YB) and the two preceding years (YB-1 and YB-2) across regions.

**
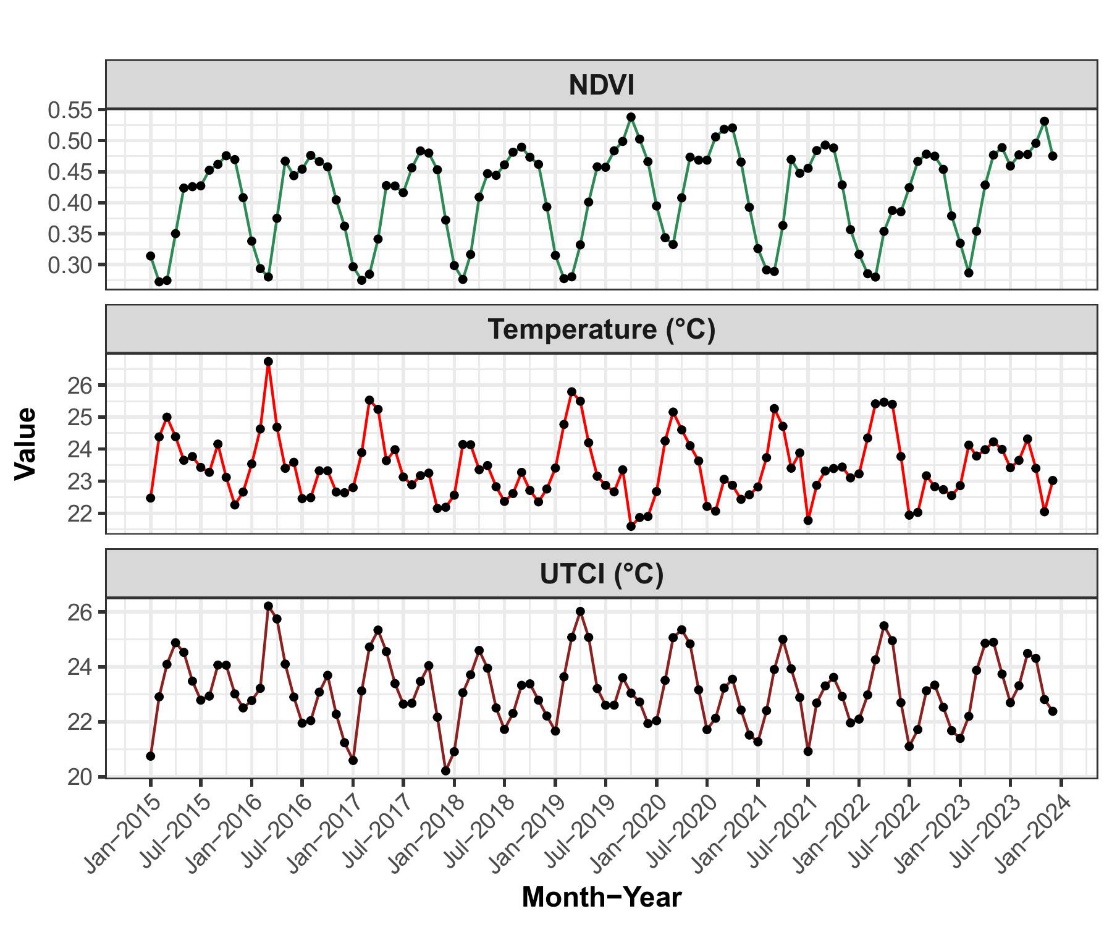
**

**Figure S4-A:** Monthly time series of mean Temperature (°C), Universal Thermal Climate Index (UTCI, °C), and Normalized Difference Vegetation Index (NDVI) in Ethiopia from 2015 to 2024. Each variable is faceted separately with independent y-axes to show seasonal and interannual variability.

*
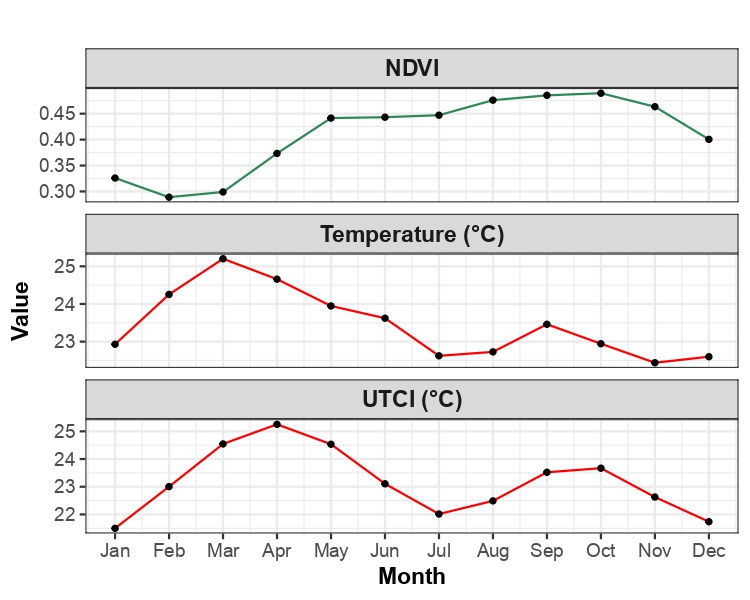
*

**Figure S4-B:** Seasonal monthly averages of Temperature (°C), Universal Thermal Climate Index (UTCI, °C), and Normalized Difference Vegetation Index (NDVI) in Ethiopia aggregated over 2015–2024. Each variable is faceted separately to highlight seasonal variability across the year.


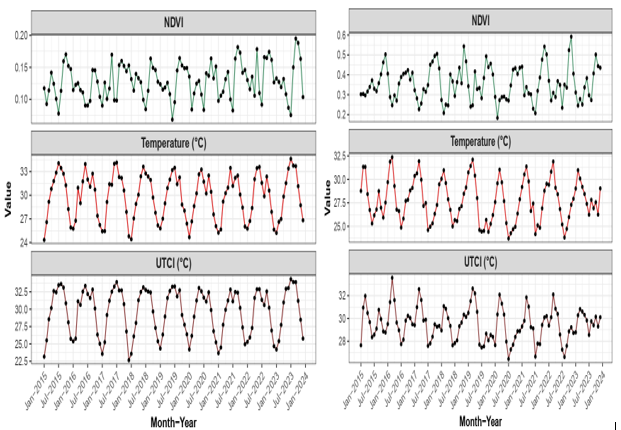


**Figure S4-C:** Monthly time series of mean Temperature (°C), Universal Thermal Climate Index (UTCI, °C), and Normalized Difference Vegetation Index (NDVI) in Afar (left panel) and Gambela (right panel) regions of Ethiopia from 2015 to 2024.


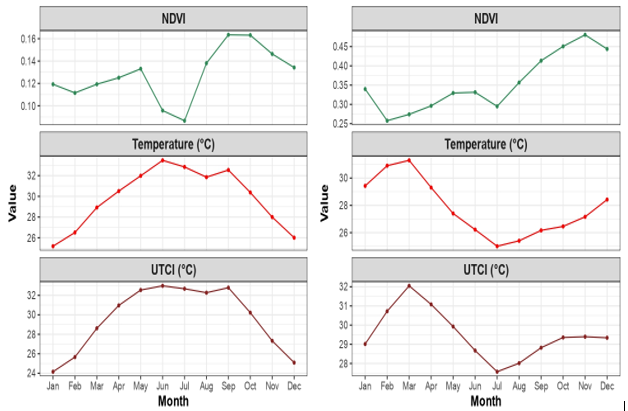


**Figure S4-D:** Seasonal monthly averages of Temperature (°C), Universal Thermal Climate Index (UTCI, °C), and Normalized Difference Vegetation Index NDVI in Afar (left panel) and Gambela (right panel) regions of Ethiopia aggregated over 2015–2024.

D)

E)

C)

F)

B)

A)

**Figure S5:** Scatter plots with local polynomial smoothing illustrating the unadjusted relationships between heat stress and vegetation exposure (x-axis) and child growth outcomes (y-axis), including stunting, wasting, and underweight (n = 7,166). Panels A–C show the relationships between heat stress (°C) and Height-for-Age Z-score (HAZ), Weight-for-Height Z-score (WHZ), and Weight-for-Age Z-score (WAZ), respectively. Panels D–F present the relationships between vegetation cover measured by the Normalized Difference Vegetation Index (NDVI) and HAZ, WHZ, and WAZ, respectively.
