# Supplementary material for: The Association Between Heat Stress and Child Stunting, Wasting, and Underweight Under Varying Vegetation Covers in Ethiopia
Source: Matern Child Nutr. 2026 Jul 28;22(3):e70227. doi: 10.1111/mcn.70227 (PMC13409331; doi:10.1111/mcn.70227)
Supplement: Supplementary file 2 — Table S1: Percent distribution of stunting, wasting and underweight among children aged 0–59 months in Ethiopia. Table S2: A descriptive summary of exposure duration to different heat stress levels across all regions during the three months preceding anthropometric data collection. Table S3: Descriptive summary of NDVI for all regions of rural residence within 3 years up to year of birth. Table S4: Adjusted associations between heat stress and diarrheal cases. Table S5: Adjusted associations between heat stress and food insecurity. [file MCN-22-e70227-s001.docx]

**Supplementary Material 1:**

**Table S1:** Percent distribution of stunting, wasting and underweight among children aged 0-59 months in Ethiopia.

|  |  | Number | | Stunting | | Wasting | | Underweight | |
| --- | --- | --- | --- | --- | --- | --- | --- | --- | --- |
|  |  | **Unweighted** | **Weighted** | **total** | **Severe** | **total** | **Severe** | **Total** | **Severe** |
| Age of Child in months | 0-5 | 1,294 | 1656006 | 17.2 | 8.1 | 22.1 | 12.2 | 13 | 4.2 |
|  | 6-23 | 2,821 | 4074423 | 37.5 | 17.5 | 12.4 | 5.5 | 19.1 | 5.9 |
|  | 24-59 | 6,273 | 8549866 | 47.1 | 22.8 | 8.5 | 3.4 | 27.1 | 8.7 |
| Region | Tigray | 791 | 831236 | 48.1 | 14.6 | 17.4 | 4.7 | 30 | 6.8 |
|  | Afar | 1,069 | 164793 | 43.5 | 23.7 | 26.3 | 12.2 | 42.5 | 19.7 |
|  | Amhara | 820 | 2799110 | 41.5 | 19.5 | 14.9 | 7.7 | 25.7 | 8.1 |
|  | Oromia | 1,204 | 6370502 | 42.2 | 20.4 | 8.4 | 4 | 22 | 6.9 |
|  | Somali | 1,283 | 794734 | 38.7 | 21.2 | 17 | 5.2 | 29.9 | 10 |
|  | Benishangul-Gumz | 728 | 127850 | 43.6 | 24.1 | 9.3 | 4.1 | 23.2 | 9.9 |
|  | SNNP | 953 | 2036518 | 41.6 | 21.2 | 9.5 | 3.7 | 20 | 6.6 |
|  | Sidama | 742 | 547484 | 35.6 | 20.1 | 14.3 | 7.1 | 20.8 | 9.8 |
|  | Gambela | 779 | 57846 | 25.1 | 11.5 | 14.8 | 6.3 | 17.6 | 6.3 |
|  | Harari | 729 | 44024 | 34.2 | 18.5 | 8.7 | 3.3 | 17.8 | 5.3 |
|  | Addis Ababa | 568 | 441915 | 13.9 | 4.8 | 3.8 | 1.9 | 5.5 | 1.1 |
|  | Dire Dawa | 722 | 64283 | 34.7 | 18 | 14.4 | 7.2 | 23.7 | 7.6 |
| Residence | Urban | 3,744 | 4162051 | 30.2 | 10.9 | 8.5 | 3.8 | 15.7 | 4.8 |
|  | Rural | 6,644 | 10118244 | 45.3 | 23.1 | 12.3 | 5.4 | 26 | 8.4 |
| Wealth quintile | Poorest | 3,146 | 3210371 | 47.2 | 23.9 | 17.3 | 7.7 | 31.6 | 12.4 |
|  | Poorer | 1,496 | 2887843 | 48 | 26.3 | 10.2 | 4 | 25.5 | 7.3 |
|  | Middle | 1,578 | 2961541 | 44.3 | 22.5 | 10 | 4.4 | 24 | 6.9 |
|  | Richer | 2,072 | 2729086 | 37 | 14.6 | 10.6 | 5.4 | 21.1 | 6.5 |
|  | Richest | 2,096 | 2491454 | 24.7 | 8.3 | 6.2 | 2.6 | 9.8 | 2.3 |
| Mother Education | No education | 5,089 | 6900050 | 46.7 | 24.6 | 12.7 | 5.7 | 28.5 | 10.2 |
|  | Primary | 3,077 | 4733156 | 39.5 | 17.2 | 10.2 | 4.5 | 20.1 | 5.4 |
|  | Secondary | 1,399 | 1677887 | 33.8 | 13.8 | 8.5 | 3.6 | 15.9 | 3.4 |
|  | More than secondary | 823 | 969203 | 18.7 | 5.5 | 9.1 | 4.7 | 10.6 | 3.3 |

**Table S2:** A descriptive summary of exposure duration (in hours) to different heat stress levels across all regions during the three months preceding anthropometric data collection.

| Region |  | Slight cold_  Stress | Nonthermal H. stress | Moderate H. stress | | Strong H. stress | Very strong H. stress | Extreme H. stress |
| --- | --- | --- | --- | --- | --- | --- | --- | --- |
| *Tigray* | Exp. (hrs) | 252 | 1242 | | 430 | 243 | 17 | 0 |
|  | Exp. (%) | 12.0 | 57.0 | | 20.0 | 11.0 | 1.0 | 0.0 |
| *Afar* | Exp. (hrs) | 0 | 865 | | 403 | 395 | **508** | **13** |
|  | Exp. (%) | 0.0 | 39.6 | | 18.5 | 18.1 | 23.3 | 0.6 |
| *Amhara* | Exp. (hrs) | 214 | 1397 | | 425 | 131 | 39 | 2 |
|  | Exp. (%) | 9.7 | 63.3 | | 19.2 | 5.9 | 1.8 | 0.1 |
| *Oromia* | Exp. (hrs) | 246 | 1462 | | 431 | 65 | 4 | 0 |
|  | Exp. (%) | 11.1 | 66.2 | | 19.5 | 2.9 | 0.2 | 0.0 |
| *Somali* | Exp. (hrs) | 43 | 1242 | | 501 | 319 | **79** | 0 |
|  | Exp. (%) | 2.0 | 56.9 | | 22.9 | 14.6 | 3.6 | 0.0 |
| *BSG* | Exp. (hrs) | 0 | 1525 | | 513 | 161 | 9 | 0 |
|  | Exp. (%) | 0.0 | 69.1 | | 23.2 | 7.3 | 0.4 | 0.0 |
| *SNNPR* | Exp. (hrs) | 37 | 1485 | | 556 | 101 | 5 | 0 |
|  | Exp. (%) | 2.0 | 68.0 | | 25.0 | 5.0 | 0.0 | 0.0 |
| *Sidama* | Exp. (hrs) | 78.9 | 1736 | | 360 | 9.39 | 0 | 0 |
|  | Exp. (%) | 3.6 | 79.5 | | 16.5 | 0.4 | 0.0 | 0.0 |
| *Gambela* | Exp. (hrs) | 0 | 1031 | | 476 | 487 | **166** | 0 |
|  | Exp. (%) | 0.0 | 47.0 | | 22.0 | 22.5 | 7.7 | 0.0 |
| *Hareri* | Exp. (hrs) | 14 | 1593 | | 518 | 59 | 0 | 0 |
|  | Exp. (%) | 0.6 | 72.9 | | 23.7 | 2.7 | 0.0 | 0.0 |
| *AA* | Exp. (hrs) | 318 | 1642 | | 224 | 0.1 | 0 | 0 |
|  | Exp. (%) | 14.6 | 75.2 | | 10.3 | 0.0 | 0.0 | 0.0 |
| *Dire dawa* | Exp. (hrs) | 282 | 1387 | | 497 | 17 | 0.9 | 0 |
|  | Exp. (%) | 12.9 | 63.5 | | 22.8 | 0.8 | 0.0 | 0.0 |

**Exp. (hrs)** refers to the maximum potential exposure duration across different heat stress categories within a 90-day period prior to the survey, measured in hours, whereas **Exp. (%)** refers to exposure duration expressed as a percentage of total hours within 90 days. BSG: *Benishangul Gumuz; AA: Addis Ababa.*

**Table S3:** Descriptive summary of NDVI for all regions of rural residence within 3 years up to the year of birth

|  |  | ***ndvi_at_birth_ (YB)*** | | | | | ***ndvi_previous_year (YB-1)*** | | | | | ***ndvi_2_years_prior_ (YB_2)*** | | | | |
| --- | --- | --- | --- | --- | --- | --- | --- | --- | --- | --- | --- | --- | --- | --- | --- | --- |
| ***Region*** | **N** | **Mean** | **SD** | **Min** | **Max** | **Mean** | | **SD** | **Min** | **Max** | **Mean** | | **SD** | **Min** | **Max** |  |
| *Afar* | 867 | 0.30 | 0.14 | 0.08 | 0.65 | 0.29 | | 0.14 | 0.08 | 0.65 | 0.28 | | 0.13 | 0.08 | 0.65 |  |
| *Amhara* | 637 | 0.47 | 0.09 | 0.24 | 0.67 | 0.47 | | 0.09 | 0.24 | 0.77 | 0.48 | | 0.09 | 0.28 | 0.77 |  |
| *Dire Dawa* | 295 | 0.40 | 0.08 | 0.23 | 0.56 | 0.40 | | 0.09 | 0.23 | 0.56 | 0.38 | | 0.09 | 0.21 | 0.55 |  |
| *Gambela* | 436 | 0.65 | 0.07 | 0.49 | 0.84 | 0.65 | | 0.07 | 0.53 | 0.84 | 0.67 | | 0.07 | 0.53 | 0.84 |  |
| *BSG* | 653 | 0.61 | 0.07 | 0.42 | 0.74 | 0.62 | | 0.07 | 0.42 | 0.75 | 0.64 | | 0.06 | 0.42 | 0.76 |  |
| *Hareri* | 433 | 0.43 | 0.06 | 0.26 | 0.56 | 0.45 | | 0.06 | 0.29 | 0.59 | 0.48 | | 0.06 | 0.31 | 0.59 |  |
| *Oromia* | 970 | 0.48 | 0.10 | 0.20 | 0.72 | 0.49 | | 0.09 | 0.20 | 0.72 | 0.50 | | 0.10 | 0.20 | 0.75 |  |
| *SNNPR* | 746 | 0.45 | 0.10 | 0.22 | 0.70 | 0.46 | | 0.10 | 0.23 | 0.74 | 0.48 | | 0.11 | 0.22 | 0.79 |  |
| *Sidama* | 561 | 0.41 | 0.09 | 0.21 | 0.68 | 0.42 | | 0.10 | 0.21 | 0.68 | 0.46 | | 0.10 | 0.22 | 0.69 |  |
| *Somali* | 1064 | 0.26 | 0.08 | 0.13 | 0.48 | 0.26 | | 0.09 | 0.13 | 0.48 | 0.25 | | 0.08 | 0.13 | 0.48 |  |
| *Tigray* | 504 | 0.40 | 0.06 | 0.27 | 0.55 | 0.39 | | 0.06 | 0.27 | 0.56 | 0.39 | | 0.06 | 0.27 | 0.56 |  |
| *National mean^†^* | 7166 | **0.44** | 0.09 | 0.25 | 0.65 | 0.45 | | 0.09 | 0.26 | 0.67 | 0.45 | | 0.09 | 0.26 | 0.67 |  |

*^†^*Signifies that the average value represents the vegetation cover (NDVI) only in rural residences. BSG: *Benishangul Gumz*

**Table S4:** Adjusted associations between heat stress and diarrheal cases

| **Adjusted Model ^†^prior to stratification** | OR (95% CI) | p-value |
| --- | --- | --- |
| No-thermal stress (≤ 26 ºC) (reference) |  |  |
| Heat stress conditions (>26 ºC) | 1.70 (1.28, 2.26) | **0.002** |
|  |  |  |
| ***Stratified analysis*** |  |  |
| Adjusted Model ^†^ for low vegetation context (NDVI < 0.44) |  |  |
| No-thermal stress (≤ 26 ºC) (reference) |  |  |
| Heat stress conditions (>26 ºC) | 1.97 (1.31, 2.95) | **0.001** |
| Adjusted Model ^†^ for moderate to high vegetation context (NDVI ≥ 0.44) |  |  |
| No-thermal stress (≤ 26 ºC) (reference) |  |  |
| Heat stress conditions (>26 ºC) | 1.43 (0.96, 2.13) | 0.07 |

^†^Adjusted for Child Age, Maternal Education, Wealth Status, and Family Size

**Table S5:** Adjusted associations between heat stress and food insecurity

| **Adjusted Model ^†^prior to stratification** | OR (95% CI) | p-value |
| --- | --- | --- |
| No-thermal stress (≤ 26 ºC) (reference) |  |  |
| Heat stress conditions (>26 ºC) | 0.78 (0.58, 1.07) | 0.12 |
|  |  |  |
| ***Stratified analysis*** |  |  |
| Adjusted Model ^†^ for low vegetation context (NDVI < 0.44) |  |  |
| No-thermal stress (≤ 26 ºC) (reference) |  |  |
| Heat stress conditions (>26 ºC) | 0.75 (0.52, 1.10) | 0.14 |
| Adjusted Model ^†^ for moderate to high vegetation context (NDVI ≥ 0.44) |  |  |
| No-thermal stress (≤ 26 ºC) (reference) |  |  |
| Heat stress conditions (>26 ºC) | 0.87 (0.52, 1.47) | 0.61 |

^†^Adjusted for Child Age, Maternal Education, Wealth Status, and Family Size
